# Primary Bedaquiline Resistance Among Cases of Drug-Resistant Tuberculosis in Taiwan

**DOI:** 10.3389/fmicb.2021.754249

**Published:** 2021-10-22

**Authors:** Sheng-Han Wu, Hsin-Hua Chan, Hseuh-Chien Hsiao, Ruwen Jou

**Affiliations:** Taiwan Centers for Disease Control, Taipei, Taiwan

**Keywords:** *Mycobacterium tuberculosis*, drug-resistant tuberculosis, bedaquiline resistance, *Rv0678*, borderline resistance

## Abstract

Bedaquiline (BDQ), which is recommended for the treatment of drug-resistant tuberculosis (DR-TB), was introduced in Taiwan in 2014. Due to the alarming emergence of BDQ resistance, we conducted BDQ resistance analyses to strengthen our DR-TB management program. This retrospective population-based study included initial *Mycobacterium tuberculosis* isolates from 898 rifampicin-resistant (RR) or multidrug-resistant (MDR) TB cases never exposed to BDQ during 2008–2019. We randomly selected 65 isolates and identified 28 isolates with BDQ MIC<0.25μg/ml and MIC≥0.25μg/ml as the control and study groups, respectively. BDQ drug susceptibility testing (DST) using the MGIT960 system and Sanger sequencing of the *atpE*, *Rv0678*, and *pepQ* genes was conducted. Notably, 18 isolates with BDQ MIC=0.25μg/ml, 38.9% (7/18), and 61.1% (11/18) isolates were MGIT-BDQ resistant and susceptible, respectively. Consequently, we recommended redefining MIC=0.25μg/ml as an intermediate-susceptible category to resolve discordance between different DST methods. Of the 93 isolates, 22 isolates were MGIT-BDQ-resistant and 77.3% (17/22) of MGIT-BDQ-resistant isolates harbored *Rv0678* mutations. After excluding 2 MGIT-BDQ-resistant isolates with borderline resistance (GU_400_growth control-GU_100_BDQ≤1day), 100% (15/15) harbored *Rv0678* gene mutations, including seven novel mutations [g-14a, Ile80Ser (*N*=2), Phe100Tyr, Ala102Val, Ins g 181–182 frameshift mutation (*N*=2), Del 11–63 frameshift mutation, and whole gene deletion (*N*=2)]. Since the other 22.7% (5/22) MGIT-BDQ-resistant isolates with borderline resistance (GU_400_growth control-GU_100_BDQ≤1day) had no mutation in three analyzed genes. For isolates with phenotypic MGIT-BDQ borderline resistance, checking for GU differences or conducting genotypic analyses are suggested for ruling out BDQ resistance. In addition, we observed favorable outcomes among patients with BDQ-resistant isolates who received BDQ-containing regimens regardless of *Rv0678* mutations. We concluded that based on MIC≥0.25μg/ml, 3.1% (28/898) of drug-resistant TB cases without BDQ exposure showed BDQ resistance, *Rv0678* was not a robust marker of BDQ resistance, and its mutations were not associated with treatment outcomes.

## Introduction

Tuberculosis (TB) and drug-resistant tuberculosis (DR-TB) are global challenges, and their prevention and control are being prioritized by the World Health Organization (WHO) (WHO, 2020a). The cure rates of drug-susceptible TB, rifampicin-resistant (RR)/multidrug-resistant tuberculosis TB (MDR-TB), and extensively drug-resistant (XDR) TB were 85, 57, and 39%, respectively (WHO, 2019a, 2020a). The higher rate of unfavorable treatment outcomes observed with DR-TB might be due to a lack of effective drugs. In addition, the current treatment regimens for DR-TB might cause severe side effects (Ginsberg and Spigelman, 2007; Jacobson et al., 2010). For better management of MDR-TB, a government-organized and hospital-based management program for cases, denoted the Taiwan MDR-TB Consortium (TMTC), was established in 2007 (Yu et al., 2015), and the treatment success rate of MDR-TB increased significantly from 61% in the pre-TMTC era to more than 82% in the TMTC era (Yu et al., 2015). Nevertheless, new drugs are still needed for the management of difficult DR-TB cases.

Bedaquiline (BDQ), a diarylquinoline, is a novel antimycobacterial drug that was approved by the United States Food and Drug Administration (FDA) in 2012. BDQ was recommended by the WHO as a core drug for the treatment of MDR and XDR-TB in 2013 (Mahajan, 2013; WHO, 2013) and is part of the WHO-endorsed, shorter, and all-oral MDR-TB regimen (WHO, 2019b, 2020b). Since BDQ was classified as the priority drug (Group A) by the WHO for the treatment of MDR-TB in 2019, 109 countries have started using BDQ to treat MDR or XDR-TB by the end of the year (WHO, 2020a). BDQ shows efficiency with improved culture conversions in DR-TB treatment (Andries et al., 2005; Diacon et al., 2014; Nguyen et al., 2016). Additionally, BDQ exhibits no cross-resistance to current first-line and second-line anti-TB drugs except clofazimine (CFZ; Andries et al., 2005). However, since the introduction of BDQ for DR-TB treatment, BDQ-resistant TB strains have gradually emerged (Andries et al., 2014; Somoskovi et al., 2015; Veziris et al., 2017). Hence, it is necessary to adopt proper drug susceptibility testing (DST) for the prescription of prompt and adequate treatment.

Studies have revealed that the mechanisms that confer BDQ resistance to *Mycobacterium tuberculosis* mainly involves three genes, namely, the *atpE* (Andries et al., 2005), *mmpR* (*Rv0678*; Hartkoorn et al., 2014; WHO, 2021), and *pepQ* genes (Almeida et al., 2016). BDQ inhibits mycobacterial ATP synthase by targeting subunit C, which is encoded by the *atpE* gene, and the AtpE protein sequence is highly conserved (Andries et al., 2005). The gene variants A63P and I66M obtained from *in vitro*-selected mutants are associated with BDQ resistance (Andries et al., 2005; Petrella et al., 2006). Isolates harboring mutations in the *atpE* gene exhibit a relatively high minimum inhibitory concentration (MIC) to BDQ (10- to 128-fold; Nguyen et al., 2018). Mutations in the *atpE* gene cause failure in the binding of BDQ to subunit C of ATP synthase and thereby maintain the transfer of hydrogen ions and ATP production (Koul et al., 2007). In addition, the transcriptional repressor of the MmpS5-MmpL5 drug export pump is encoded by the *Rv0678* gene (Andries et al., 2014). Mutations in *Rv0678* cause upregulation of MmpS5-MmpL5 expression and the export of BDQ (Andries et al., 2014). Nevertheless, *Rv0678* mutations are associated with low-level cross-resistance between BDQ and CFZ (Somoskovi et al., 2015) and lead to 2- to 8-fold increases in the MICs of BDQ and CFZ (Andries et al., 2014). Notably, a previous study highlighted that resistance to azole antifungal drugs is associated with *Rv0678* mutations causing upregulation of the MmpS5-MmpL5 efflux pump (Milano et al., 2009). In addition, mutations in the *pepQ* gene, which encodes aminopeptidase, are associated with low-level BDQ and CFZ resistance (Almeida et al., 2016).

BDQ was introduced in Taiwan in 2014 for the treatment of DR-TB. Due to the alarming emergence of BDQ resistance, we established an algorithm for detecting BDQ resistance in our programmatic management of drug-resistant TB (PMDT) programs. In this study, we performed BDQ susceptibility testing and depicted the extent of BDQ resistance in DR-TB cases.

## Materials and Methods

### Study Design and Isolates

**Figure 1:**
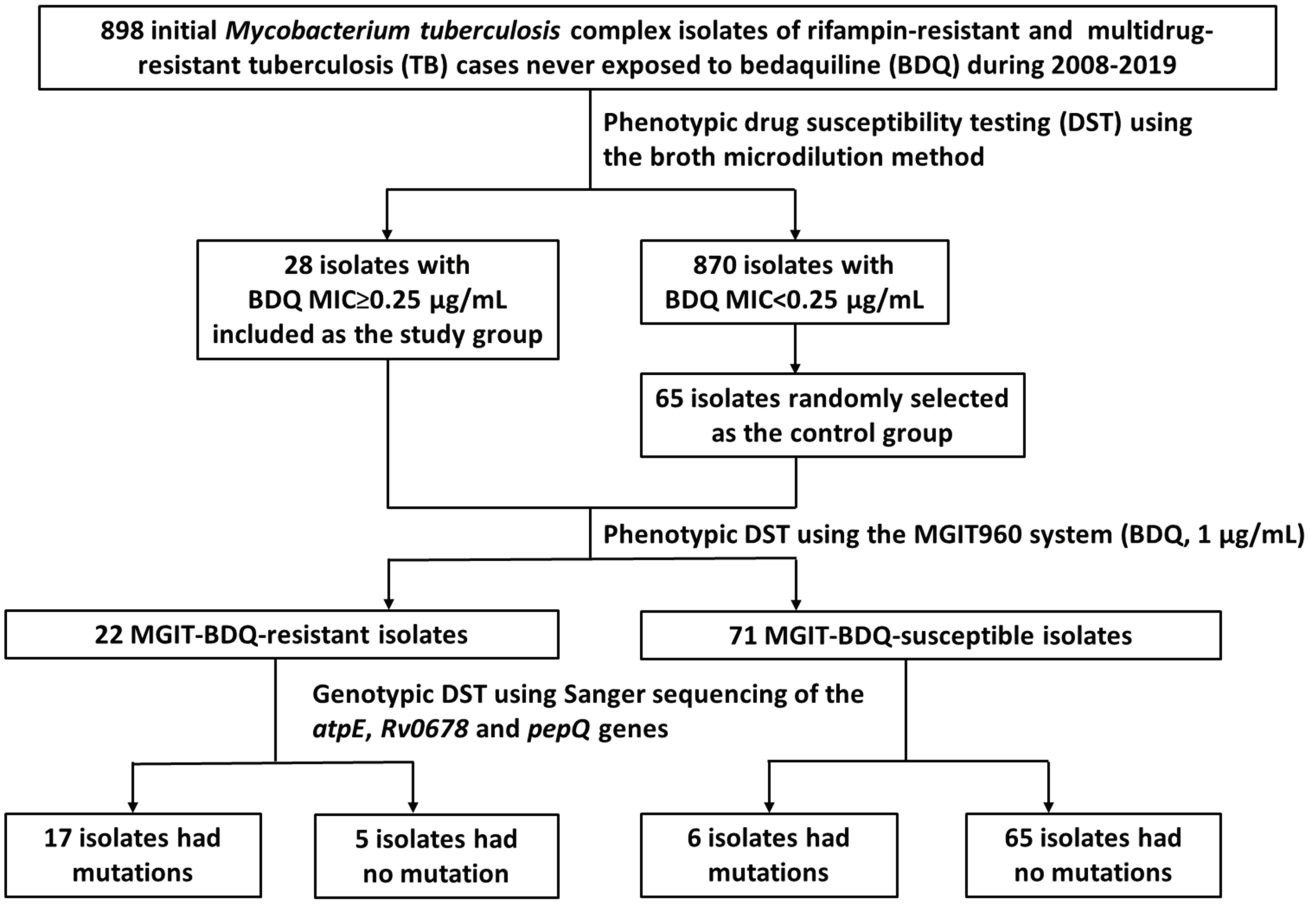
A flowchart of study isolates selection process.

This retrospective population-based study included initial *M. tuberculosis* complex isolates from 898 RR- and MDR-TB cases never exposed to BDQ during 2008–2019. Universal DST for culture-positive *M. tuberculosis* isolates was implemented in Taiwan. We conducted broth microdilution (BMD) method to determine MICs of initial isolates of RR- and MDR-TB cases confirmed from 2008 to 2019. The primary BDQ resistance rate was calculated using total number of initial isolates of RR- and MDR-TB cases as the denominator and the number of BDQ-resistant isolates with MIC≥0.25μg/ml as the numerator (Figure 1). We randomly selected 65 isolates with BDQ MIC<0.25μg/ml as the control group and 28 isolates with MIC≥0.25μg/ml as the study group. The characterizations and treatment outcomes of the cases were obtained from the National TB Registry.

This study was approved by the Institutional Review Board of Centers for Disease Control, Ministry of Health and Welfare (TwCDC IRB No. 109205) and analyzed only archived *M. tuberculosis* isolates, and thus, written informed consent from the participants was waived. Cultivation and processing of *M. tuberculosis* were performed in a certified biosafety level three laboratory. All methods were performed in accordance with the relevant guidelines and regulations.

### Phenotypic DST

*M. tuberculosis* isolates were subjected to DST using the agar proportion method with 7H10 and 7H11 medium (Becton, Dickinson and Company, Spark, MD, United States). Drug resistance was defined as the growth of 1% of colonies in a drug-containing medium. According to WHO recommendations, the critical concentrations of the tested drugs in 7H10 medium were the following: rifampicin (RIF), 1μg/ml; isoniazid (INH), 0.2μg/ml; ethambutol (EMB), 5μg/ml; streptomycin (SM), 2μg/ml; moxifloxacin (MFX), 0.5μg/ml; and levofloxacin (LFX), 1μg/ml (WHO, 2018). The critical concentrations of the tested drugs in 7H11 medium were the following: rifabutin (RFB), 0.5μg/ml; kanamycin (KM), 6μg/ml; amikacin (AMK), 6μg/ml; capreomycin (CM), 10μg/ml; ethionamide (ETO), 10μg/ml; para-aminosalicylic acid (PAS), 8.0μg/ml; and cycloserine (CS), 60μg/ml (CLSI, 2018; WHO, 2018). The resistance to pyrazinamide (PZA, 100μg/ml) and BDQ (1μg/ml) was tested using Bactec MGIT 960 as described previously (WHO, 2018). The growth on the control medium was compared to that on the drug-containing medium to determine susceptibility. The DST results were categorized as resistant or susceptible, and the tests were validated by determining the susceptibility of *M. tuberculosis* H37Rv. MDR is defined as an *M. tuberculosis* isolate resistant to at least INH and RIF. Pre-XDR is defined as an MDR isolate resistant to either fluoroquinolones (FQs; pre-XDR-FQs) or at least one of the injectable drugs (pre-XDR-INJ). XDR is defined as an MDR isolate resistant to a FQ and at least one of the injectable drugs.

Phenotypic MIC testing was performed according to previously described methods (Kaniga et al., 2016). The MIC plate contained 12 antimicrobial agents, namely, RIF, INH, EMB, LFX, MFX, ofloxacin (OFX), KM, AMK, CAP, BDQ, CFZ, and LZD. The H37Rv strain was included in each test as the control, and the results were interpreted by two independent readers. The interpretive criterion for BDQ resistance was MIC≥0.25μg/ml (Kaniga et al., 2020).

### Genotypic DST

One loop (0.5μl) of bacteria was placed into a microtube and resuspended in 500μl of Tris-EDTA buffer. The bacterial liquid was inactivated at 95°C for 20min. The bacterial lysate was centrifuged at 12,000×g for 1min, and the supernatant was used as a template for PCR. In this study, we analyzed three BDQ resistance-associated genes, namely, *atpE*, *Rv0678*, and *pepQ*. The specific primers were designed based on *M. tuberculosis* strain H37Rv (GenBank: AL123456.3) to amplify the whole genes by PCR (Table 1). PCRs were performed using a HotStarTaq Master Mix kit (QIAGEN, Germany). Each reaction mixture contained 12.5μl of 2×HotStarTaq Master Mix (QIAGEN, Germany), 0.5μl of each primer (10μm), and 2–5μl of bacterial lysate. Double-distilled water was added to the mixture to obtain a total volume of 25μl. The PCR conditions were as follows: hot start at 95°C for 10min; 35cycles of 95°C for 1min; 56–64°C (according to the optimal primer annealing temperature) for 1min; and 72°C for 1min; and a final elongation step of 72°C for 5min. The PCR products were analyzed using the capillary electrophoresis QIAxcel Advanced system (QIAGEN, Germany). The DNA sequence was confirmed by Sanger sequencing (Genomics BioSci & Tech, Taiwan). In addition, sequence assembly and mutation identification were performed using Sequencher (Gene Codes Corporation, United States) and Molecular Evolutionary Genetics Analysis 10 (MEGA 10) software.

**Table 1:** PCR primers, Tm, and amplicon size of BDQ resistance-associated genes.

Primer	Sequence (5' to 3')	Tm (°C)	Amplicon size (bp)
*atpE*-F	CCA AGC GAT GGA GCT CGA AGA GG	58	439
*atpE*-R	GGG AAT GAG GAA GTT GCT GGA CTC G	58	439
*Rv0678*-F	GCT TGA GAG TTC CAA TCA T	56	674
*Rv0678*-R	CGC ATC AAC AAG GAG TGA	56	674
*Rv0678*-2F	CAA CCA GGA TGA GCA GCG GTA TCC	60	1,145
*Rv0678*-2R	CGG TTG GCG ACC TTT GCT CTG G	60	1,145
*pepQ*-1F	GAA CAG GCG GAG AAC CAC CAT CG	58	768
*pepQ*-1R	GGC GCC GAA GTC GAT CTT CAC G	58	768
*pepQ*-2F	TGA TGC TCG ATC ATG GCG CTG ACG	64	689
*pepQ*-2R	CTT GCC CGG TTT GAC GTG CTG G	64	689

### Genotyping

Spacer oligonucleotide typing (spoligotyping) analysis was used for genotyping. A commercially available kit (Isogen Bioscience BV, Maarssen, Netherlands) was used as described previously (Kamerbeek et al., 1997). Briefly, the amplified DNA was hybridized onto a membrane that was covalently precoated with a set of 43 spacer oligonucleotides derived from the spacer sequences of *M. tuberculosis* H37Rv and *M. bovis* P3. The ECL® Detection system (GE Healthcare, United States) was used for the final image detection. The spoligotypes were compared with the SITVIT global database.^1^

### Statistical Analyses

The chi-squared test or Fisher’s exact test (when expected cell size <5) was used for the univariate analysis of categorical variables. A value of *p*<0.05 was considered to indicate statistical significance. Odds ratios (ORs) and 95% confidence intervals (CIs) were calculated to estimate the correlation between the BDQ MIC and variables.

## Results

### Characteristics of the Study Population

Of the 898TB cases, 72.8% (654/898) were male patients, and 81.6% (733/898) and 18.4% (165/898) were new and previous TB cases, respectively (Table 2). The median age of the patients with RR/MDR-TB was 58.0years (IQR: 45.0–72.0). According to the AFB smear results, 57.1% (513/898) were smear-positive cases, and this value was higher than the proportion of general TB cases (38.7%; *p*<0.001; Chiang et al., 2018). Chest radiography of 24.2% (217/898) of cases showed cavitation, and 87.1% (782/898) and 7.0% (63/898) of the cases were pulmonary TB cases and had pleural effusion, respectively. Of the 898*M. tuberculosis* isolates, 486 (54.1%) cases had Beijing family genotypes, and this number is significantly higher than that of the general TB cases (44.4%; *p*<0.01; Jou et al., 2005). Based on the DST results, 202 (22.5%), 608 (67.7%), 74 (8.2%), and 14 (1.6%) cases were classified as RR-TB, MDR-TB, pre-XDR-TB, and XDR-TB, respectively. In addition, a BDQ MIC≥0.25μg/ml was not associated with sex, age, treatment history, drug resistance profiles, or genotypes.

**Table 2:** Demographic and clinical characteristics of 898 tuberculosis cases.

Characteristics	No. (%) of isolates	No. of isolates BDQ MIC≥0.25μg/ml (3.1%, 28/898)	No. of isolates BDQ MIC <0.25μg/ml (96.9%, 870/898)	Univariate analysis		
				OR	95% CI	*p* value
**Gender**
Male	654 (72.8)	18	636	0.66	0.30–1.46	0.30
Female	244 (27.2)	10	234	Ref.		
**Age**
≤25	53 (5.9)	1	52	0.58	0.85–4.37	0.72^#^
26–44	164 (18.3)	5	159	0.97	0.36–2.60	1.00
45–64	349 (38.9)	11	338	1.02	0.47–2.20	1.00
≥65	332 (37.0)	11	321	1.11	0.51–2.39	0.79
**Case category**
New	733 (81.6)	23	710	1.04	0.39–2.77	0.92
Previously treated	165 (18.4)	5	160	Ref.		
**AFB smear**
Positive	513 (57.1)	19	494	1.61	0.72–3.59	0.24
Negative	348 (38.8)	8	340	0.62	0.27–1.43	0.26
Unknown	37 (4.1)	1	36	0.86	0.11–6.49	1.00^#^
**Chest radiography**
Normal	20 (2.2)	1	19	1.66	0.21–12.85	1.00^#^
Abnormal with cavitation	217 (24.2)	3	214	0.37	0.11–1.23	0.12^#^
Abnormal without cavitation	643 (71.6)	24	619	2.43	0.84–7.08	0.13^#^
Abnormal but not related to TB	15 (1.7)	0	15	NA	NA	1.00^#^
Unknown	3 (0.3)	0	3	NA	NA	1.00^#^
**Site of tuberculosis**
Pulmonary	782 (87.1)	24	758	0.89	0.30–2.60	1.00^#^
Extrapulmonary	116 (12.9)	4	112	Ref.		
**Pleural effusion**
Yes	63 (7.0)	2	61	Ref.		
No	835 (93.0)	26	809	0.98	0.23–4.23	1.00^#^
**Genotype**
Genotype Beijing family	486 (54.1)	15	471	0.98	0.46–2.08	1.00
Non-Beijing family	412 (45.9)	13	399	Ref.		
**Drug resistance pattern**						
RR	202 (22.5)	5	197	0.74	0.28–1.98	0.55
MDR	608 (67.7)	20	588	1.20	0.52–2.76	0.67
Pre-XDR	74 (8.2)	3	71	1.35	0.40–4.58	0.72^#^
XDR	14 (1.6)	0	14	NA	NA	1.00^#^

AFB smear, acid-fast bacilli smear; RR, rifampicin-resistant; MDR, multidrug-resistant; Pre-XDR, Pre-extensively drug-resistant; XDR, extensively drug-resistant; OR, odds ratio; CI, confidence interval; Ref., reference; and NA, not applicable due to a small no. of cases.

# Fisher’s exact probability test (two tailed).

### BDQ Susceptibility

#### Phenotypic DST

**Figure 2:**
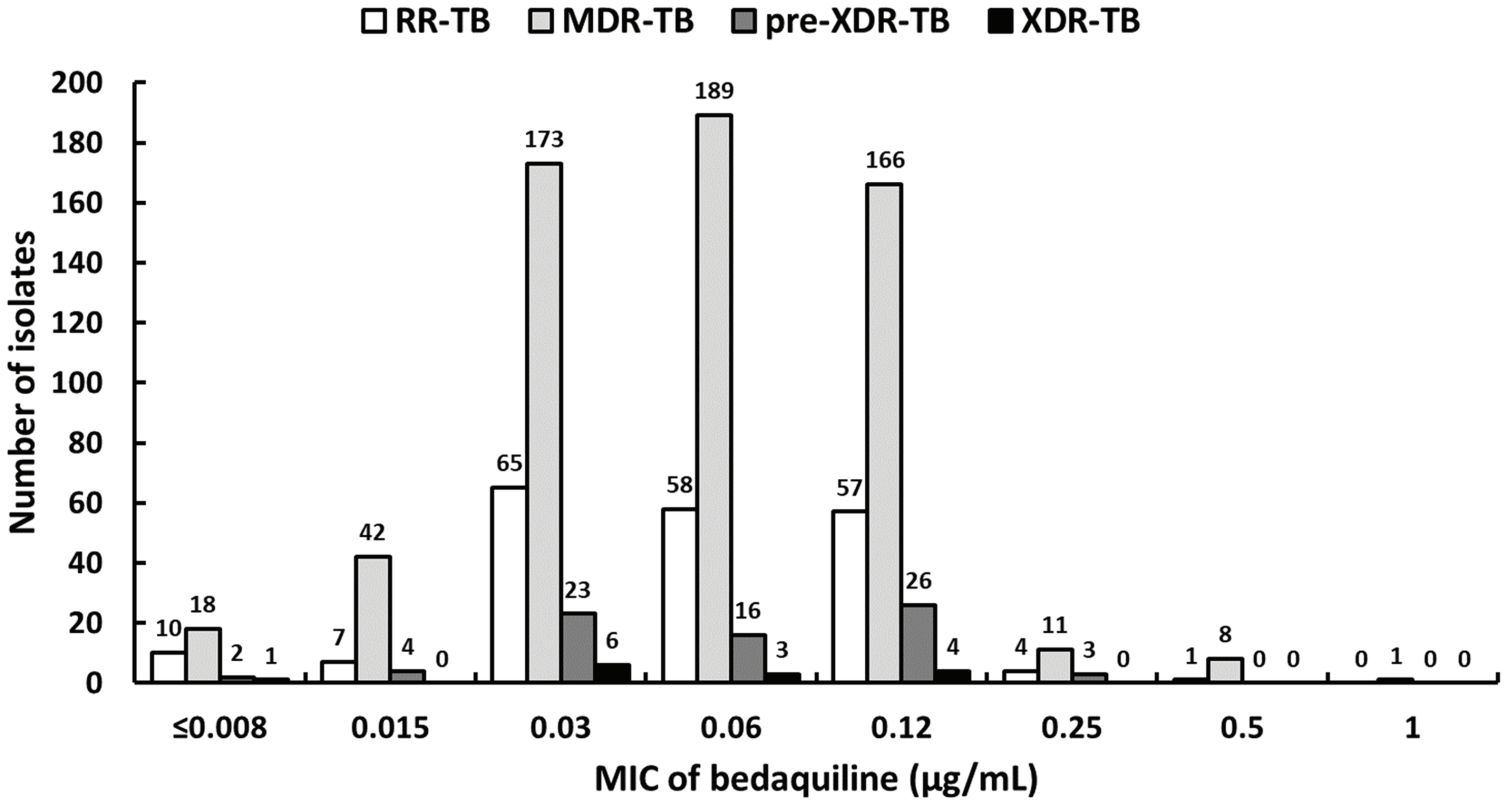
Distributions of the bedaquiline MIC values among 898*Mycobacterium tuberculosis* complex isolates including rifampicin-resistant (RR), multidrug-resistant (MDR), pre-extensively drug-resistant (pre-XDR), and extensively drug-resistant XDR isolates.

The BDQ MIC_50,_ MIC_90,_ and MIC_99_ values of the 898 isolates were 0.06, 0.12, and 0.5μg/ml, respectively (Figure 2). We observed a unimodal BDQ MIC distribution that peaked at 0.03 (29.7%; Figure 2). Based on the resistance breakpoint (≥ 0.25μg/ml), we found 3.1% (28/898) isolates were resistant to BDQ. Of the 28 isolates with BDQ MIC≥0.25μg/ml, 2.5% (5/202), 3.3% (20/608), 4.1% (3/74), and 0.0% (0/14) cases were classified as RR-TB, MDR-TB, Pre-XDR-TB, and XDR-TB, respectively (Table 2). However, a study conducted in China, the highest BDQ resistance was found in XDR-TB (16.7%), followed by MDR-TB (5.6%) and Pre-XDR-TB (4.2%; Yang et al., 2020).

**Figure 3:**
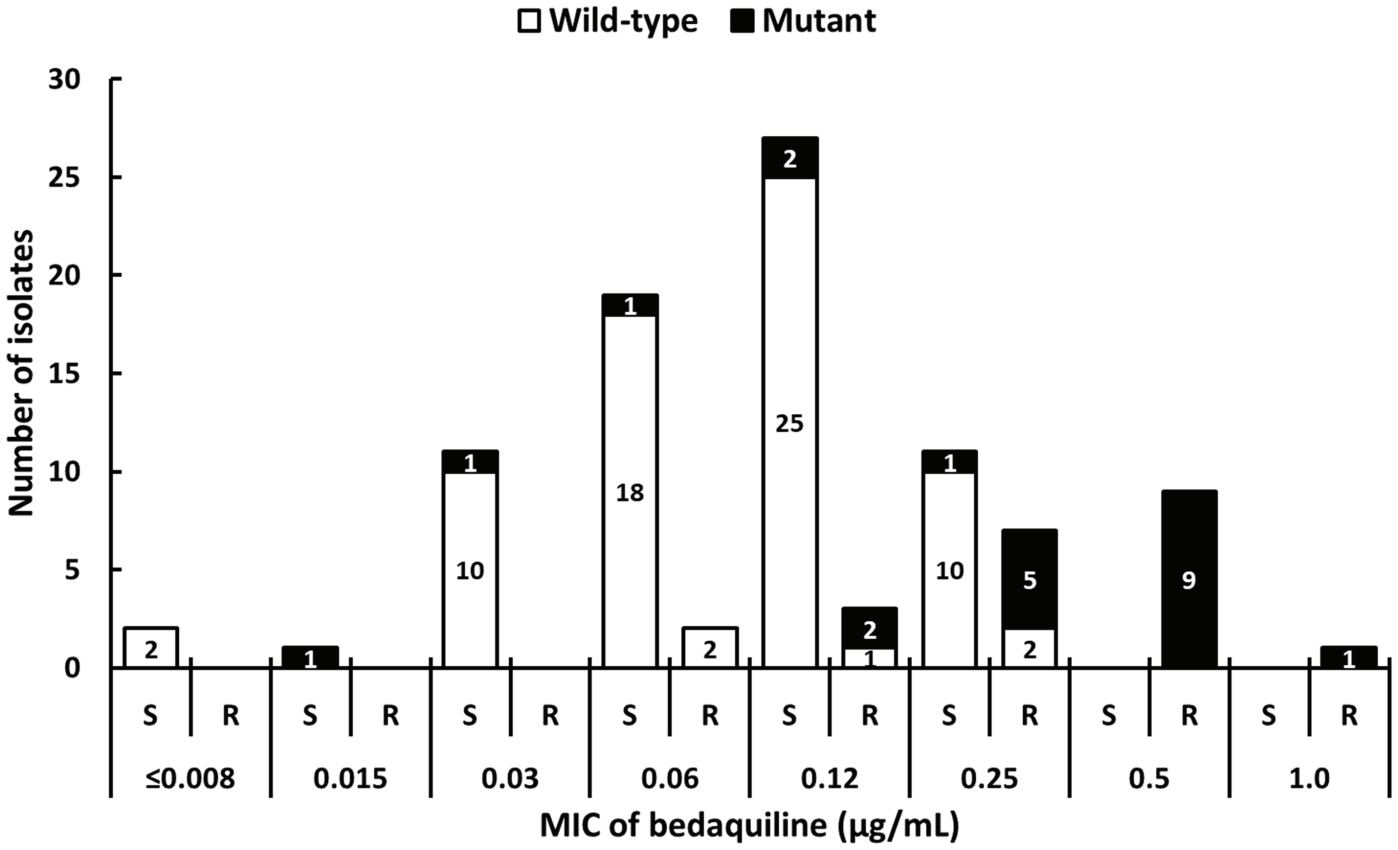
Distributions of the bedaquiline MIC values and gene mutations of 93 study isolates. R, resistant; S, susceptible.

The phenotypic drug resistance profiles and MIC distribution of 93 isolates are shown in Supplementary Table S1 and Figure 3, respectively. Of the 93 isolates, 28 isolates had MIC≥0.25μg/ml, 22 (23.7%) isolates exhibited MGIT-BDQ resistance, and 23 (24.7%) isolates harbored mutations in the *atpE*, *Rv0678*, and *pepQ* genes (Figure 3 and Table 3). Two silent mutations, *atpE* E61E and *pepQ* A210A, were observed in 2 MGIT-BDQ-susceptible isolates with MIC<0.25μg/ml. The ranges of the BDQ MICs obtained for isolates with wild-type (WT) and *Rv0678* mutations were ≤0.008 to 0.25μg/ml and 0.015 to 1μg/ml, respectively (Table 3). We found that 10 MGIT-BDQ-susceptible and genotypic WT isolates had MICs close to the critical concentration (0.25μg/ml; Figure 3). Interestingly, four isolates showed whole *Rv0678* gene deletion (Del 778,989–779,851), one and two isolates had MIC values of 0.5μg/ml and 0.25μg/ml, respectively, and one genotypic hetero-resistant isolate, Del 778,989–779,851 + WT, exhibited a MIC value equal to 0.12μg/ml (Table 3).

**Table 3:** Genotypic and phenotypic BDQ drug susceptibility testing results of 93 study isolates.

Genotypic DST (Mutation)			Phenotypic DST (No. of isolates)										
*atpE*	*pepQ*	*Rv0678*	No of isolates (%)	MGIT 1.0μg/ml		MIC (μg/ml)							
				S	R	0.008	0.015	0.03	0.06	0.12	0.25	0.5	1
WT	WT	WT	70 (75.3)	65	5^*^	2		10	20	26	12		
E61E	WT	WT	1 (1.1)	1	0				1				
WT	A210A	WT	1 (1.1)	1	0					1			
WT	WT	c-11a	1 (1.1)	1	0		1						
WT	WT	g-14a	1 (1.1)	0	1							1	
WT	WT	C46Y	1 (1.1)	0	1					1			
WT	WT	S53P	2 (2.2)	0	2							1	1
WT	WT	I80S	3 (3.2)	0	3					1	2		
WT	WT	L83P	1 (1.1)	0	1						1		
WT	WT	G87A	1 (1.1)	1	0			1					
WT	WT	F100Y	1 (1.1)	0	1							1	
WT	WT	A102V	1 (1.1)	0	1							1	
WT	WT	L114P	1 (1.1)	0	1							1	
WT	WT	Del 11–63 Fs (29 stop)	1 (1.1)	0	1							1	
WT	WT	Ins g 181–182 Fs (80 stop)	2 (2.2)	0	2							2	
WT	WT	Ins a 274–275 Fs (92 stop) + WT	1 (1.1)	0	1						1		
WT	WT	Whole gene Del + WT	1 (1.1)	1^#^	0					1			
WT	WT	Whole gene Del	3 (3.2)	1^^^	2						2	1	

DST, drug susceptibility testing; WT, wild type; Del, deletion; Ins, insertion; and Fs, frameshift mutation.

* Borderline resistance: GU_400_ growth control – GU_100_ BDQ≤1day.

# Borderline susceptible: GU_100_ BDQ – GU_400_ growth control ≤1day^#^.

^ Borderline susceptible: GU_100_ BDQ – GU_400_ growth control ≤2day^.

Notably, we found 5 MGIT-BDQ broadline-resistant isolates with discordant genotypic WT results, and the time lag between the MGIT GU_400_ growth control and GU_100_ experimental groups was less than 1day. Furthermore, among the 2 MGIT-BDQ-susceptible isolates with whole *Rv0678* gene deletion, one isolate showed a MIC equal to 0.12μg/ml, and the lag time between GU_400_ and GU_100_ was less than 1day; the other isolate presented a MIC of 0.25μg/ml, and the lag time between GU_400_ and GU_100_ was less than 2days (Table 3).

Of the 18 isolates with BDQ MIC=0.25μg/ml, 38.9% (7/18), and 61.1% (11/18) isolates were MGIT-BDQ resistant and susceptible, respectively (Figure 3). It might not be appropriate to use a single breakpoint to determine categorical DST results. Therefore, we recommended to define MIC=0.25μg/ml as intermediate susceptible to resolve discordant results obtained from different DST methods.

#### Genotypic DST

Of the 23 isolates harboring mutations in the *atpE*, *Rv0678*, or *pepQ* genes, six (26.1%) isolates were MGIT-BDQ susceptible. Of these, two isolates harbored silent mutations in the *atpE* and *pepQ* genes (each isolate had a mutation in one of these genes), and four isolates harbored mutations in *Rv0678*, including c-11a (*N*=1, Beijing), G87A (*N*=1, T1), and whole gene deletion (*N*=2, Beijing; Table 3). In addition, 17 (73.9%) MGIT-BDQ-resistant isolates harbored *Rv0678* mutations, including five known mutations (*N*=6), namely, C46Y (*N*=1, T2), S53P (*N*=2, 2 EAI), L83P (*N*=1, Beijing), L114P (*N*=1, Beijing), and Ins a 274–275/Fs (*N*=1, Haarlem-3), and seven novel mutations (*N*=11; Table 3). The analysis of these novel mutations showed that six isolates harbored mutations, namely, g-14a (*N*=1, EAI), I80S (*N*=3, Haarlem-3), F100Y (*N*=1, unidentified), and A102V (*N*=1, T2), two isolates exhibited whole *Rv0678* gene deletion (Beijing), and three isolates harbored Del 11–63/Fs (29 stop; *N*=1, T1) and Ins g 181–182/Fs (80 stop; *N*=2, unidentified) mutations (Table 3). Two hetero-resistant isolates, Ins a 274–275/Fs (92 stop) + WT (Haarlem-3) and whole *Rv0678* gene deletion + WT (Beijing), were identified.

### Characteristics and Clinical Outcomes of Cases With MGIT-BDQ-Resistant Isolates

The mean age of the 22 patients with MGIT-BDQ-resistant isolates was 54.5±16.0years, 77.3% (17/22) of the patients were male, and 86.4% (19/22) were new cases (Supplementary Table S2). These 22 cases included 2 (9.1%) cases of RR-TB, 15 (68.2%) cases of MDR-TB, four (18.2%) cases of Pre-XDR-TB, and one (4.5%) case of XDR-TB. The major spoligotypes of the 22 isolates were 31.8% (7/22) Beijing family, 27.3 (6/22) Haarlem, and 13.6% (3/22) East African-Indian (EAI), respectively. We found isolates with identical *Rv0678* mutation had the same spoligotypes (Supplementary Table S2). Of the 14 (63.6%) cases with favorable treatment outcomes, regardless of the *Rv0678* mutations, five cases were treated with BDQ-containing regimens, and these included one case still under treatment with sputum culture conversion.

## Discussion

BDQ is a core drug in the treatment of DR-TB responsible for reducing mortality and improving outcomes (Olayanju et al., 2018; Schnippel et al., 2018; Mbuagbaw et al., 2019). The emergence of BDQ resistance raises concerns in the DR-TB control program. In this retrospective population-based study, the rate of BDQ resistance (MIC≥0.25μg/ml) among DR-TB cases without BDQ and CFZ exposure was found to be 3.1% (28/898) in Taiwan, whereas other studies found values of 1.0% in France (Veziris et al., 2017), 1.3% in Russia (Peretokina et al., 2020), 2.2–3.9% in China (Pang et al., 2017; Liu et al., 2020; Yang et al., 2020), and 2.3% in a multicountry population (Diacon et al., 2014; Pym et al., 2016; Villellas et al., 2017). Furthermore, we identified 77.3% (17/22) of MGIT-BDQ-resistant isolates harboring *Rv0678* mutations (Supplementary Table S2), which might not be associated with CFZ cross-resistance (Xu et al., 2017; Ghodousi et al., 2019; Beckert et al., 2020), whereas the other studies found corresponding values of 50.0–66.7% in China (Pang et al., 2017; Yang et al., 2020), 66.7% in Australia (Martinez et al., 2018), 71.4% in Germany (Andres et al., 2020), 75.0% in France (Veziris et al., 2017), 100% in South Africa (Nimmo et al., 2020b), 100% in Russia (Zimenkov et al., 2017; Peretokina et al., 2020), and 100% in a multicountry population (Villellas et al., 2017). BDQ resistance might naturally occurred or during treatment with other anti-TB drugs (Yang et al., 2020) or previous use of antifungal drugs (Milano et al., 2009; Hartkoorn et al., 2014). In Taiwan, the 4% prevalence rate of azole-resistant *Aspergillus fumigatus* clinical isolates mainly emerged from the environment and during antifungal treatment (Wu et al., 2020), and its influence on BDQ resistance remains elusive. The reason for preexisting BDQ resistance is unknown and challenges the future use of BDQ in DR-TB treatment.

Notably, BDQ resistance determined using by MGIT DST using the suggested that a≥1day cutoff value might yield disputable susceptibility results. We observed that 22.7% (5/22) of MGIT-BDQ-resistant isolates had no mutations in the *atpE*, *Rv0678*, *pepQ* genes, and the same observations were obtained in France, China, and Iran (Pang et al., 2017; Veziris et al., 2017; Ghajavand et al., 2019; Liu et al., 2020). Due to the identification of 5 MGIT-BDQ broadline-resistant isolates with genotypic WT results for *Rv0678*, the assessment of raw MGIT-BDQ DST data is suggested. Nevertheless, MGIT-BDQ-resistant isolates with no mutations in the *atpE*, *Rv0678*, and *pepQ* genes might be caused by other resistance mechanisms, such as non-Rv0678 transcriptional regulators of mmpL5/mmpS5, as proven in a system consisting of two components, TrcR and TrcS, using whole-genome microarray technology (Wernisch et al., 2003), or overexpression of the BDQ-response regulons *Rv0324* and *Rv0880* (Peterson et al., 2016).

Excluding the five aforementioned isolates, the susceptibility to BDQ determined using the WHO interim critical concentration for MGIT (1μg/ml) was reliable (WHO, 2018). Nevertheless, we found that an MIC of 0.5μg/ml, but not 0.25μg/ml, could consistently determine BDQ resistance based on mutations in *Rv0678* (Table 3). Consequently, an MIC of 0.25μg/ml could be considered an intermediate-susceptible category for DST as defined by the Clinical Laboratory Standards Institute.

Previous studies have revealed that mutations scattered across *Rv0678* result in MIC shifts and might not be linked to specific *M. tuberculosis* lineages (Villellas et al., 2017; Zimenkov et al., 2017; Ismail et al., 2019; Battaglia et al., 2020; Peretokina et al., 2020; Nimmo et al., 2020b). In this study, *Rv0678* mutations were not associated with specific genotypes. Of note, we identified four Beijing isolates with whole *Rv0678* gene deletion that exhibited various MICs ranging from 0.012 to 0.5μg/ml, and whether this deletion is a lost-of-function mutation or due to an existing epistatic factor merits further investigation. The intergenic region mutation c-11a (MIC=0.015μg/ml), which is found exclusively in Beijing isolates, was consistent with that observed in a study conducted in Belgium; however, it might not be associated with drug resistance (Villellas et al., 2017). Because isolates harboring the G87R mutation in *Rv0678* are susceptible to BDQ (Martinez et al., 2018; Battaglia et al., 2020), a novel G87A mutation (MIC=0.03μg/ml) identified in this study might not have impacted the structure and stability of the protein. Studies have revealed that mutations occurring at amino acids 62–68 interfere with helix recognition of the DNA-binding domain in other MarR family regulators (Hong et al., 2005). We found that in 2 BDQ-resistant isolates, the introduction of Del 11–63/Fs (29 stops; *N*=1) and Ins g 181–182/Fs (80 stops) might cause loss of the functional folded protein and subsequently destabilize Rv0678 (Kadura et al., 2020). The association of drug resistance and novel *Rv0678* mutations found in MGIT-BDQ-resistant isolates merits further investigation.

Furthermore, resistance-conferring mutations in the *atpE* gene and other probable BDQ resistance-associated genes, *pepQ*, *Rv1979c* (Ismail et al., 2018), and *mmpL5*, might be potential determinants. Because *atpE* and *pepQ* mutations were found to not confer high- or low-level BDQ resistance in this study and the existing *Rv0678* mutations might not be associated with BDQ resistance, careful evaluation of the prescription of BDQ in the regimens for DR-TB treatment is recommended. In our PMDT program, periodical expert consultation on treatment and management is conducted through the TMTC.

Because most *Rv0678* mutations are associated with low-level BDQ resistance (Veziris et al., 2017; Villellas et al., 2017; Xu et al., 2017; Zimenkov et al., 2017; Battaglia et al., 2020; Peretokina et al., 2020; Yang et al., 2020), scarce studies have investigated their impact on treatment outcomes. A study using a mice model showed that BDQ still exhibits bactericidal activity against isolates with *Rv0678* mutations and activity lower than that found in the absence of *Rv0678* mutations (Andries et al., 2014). Of the five cases with *Rv0678* mutations that have not been exposed to BDQ and CFZ in South Africa, two cases exhibited favorable outcomes after treatment with BDQ-containing regimens (Nimmo et al., 2020a). Of the five patients who acquired BDQ resistance with *Rv0678* mutations after BDQ treatment, four had unfavorable outcomes (Nimmo et al., 2020a). A study conducted in China showed that two BDQ- or CFZ treatment-naïve cases with *Rv0678* mutations showed favorable outcomes after BDQ treatment (Liu et al., 2020). Nevertheless, of the five cases that acquired BDQ resistance with *Rv0678* mutations after BDQ treatment, three cases had unfavorable outcomes (Liu et al., 2020). This was a retrospective cohort study, and we report observed results. Individualized regimens for 22 MGIT-BDQ-resistant TB cases were in Supplementary Table S3. Nevertheless, in line with WHO recommendations, we observed that DR-TB cases with isolates harbored *Rv0678* mutations could be treated with nonBDQ-containing regimens of at least four drugs, and had favorable outcomes (Supplementary Table S3). Since the frequencies of favorable outcomes in other studies with limited numbers of TB cases, our collective results provide insights for DR-TB management. Particularly, of the five primary BDQ-resistant cases treated with a regime that included BDQ, four cases had favorable outcomes, and one was under treatment with sputum culture conversion (Supplementary Table S2). No case of relapse was recorded after 7years of BDQ use.

## Conclusion

This study provides the first report on the population-based surveillance and molecular characteristics of BDQ susceptibility among DR *M. tuberculosis* isolates in Taiwan. For isolates with borderline phenotypic resistance to MGIT-BDQ, checking for GU differences or conducting genotypic analyses are suggested to rule out the possibility of BDQ resistance. In accordance with other previous studies, the present study found that BDQ resistance was mainly caused by *Rv0678* mutations, including some that were not resistance-conferring mutations. Furthermore, a MIC of 0.25μg/ml could be considered an intermediate-susceptible category for DST. We observed favorable outcomes among patients with DR-TB receiving BDQ-containing regimens regardless of *Rv0678* mutations. In the PMDT program, comprehensive DST should be performed to inform the prescription of BDQ in the treatment of DR-TB with the aim of achieving better treatment outcomes.

## Data Availability Statement

The raw data supporting the conclusions of this article will be made available by the authors, without undue reservation.

## Author Contributions

RJ designed the research. S-HW, C-HC, and H-CH performed the experiments. RJ and S-HW analyzed the results and wrote the manuscript. All authors contributed to the article and approved the submitted version.

## Funding

This study was supported by grants (MOHW109-CDC-C-315-114403 and MOHW110-CDC-C-315-114405) from the Taiwan Centers for Disease Control, Ministry of Health and Welfare, Taiwan.

## Conflict of Interest

The authors declare that the research was conducted in the absence of any commercial or financial relationships that could be construed as a potential conflict of interest.

## Publisher’s Note

All claims expressed in this article are solely those of the authors and do not necessarily represent those of their affiliated organizations, or those of the publisher, the editors and the reviewers. Any product that may be evaluated in this article, or claim that may be made by its manufacturer, is not guaranteed or endorsed by the publisher.

## Footnotes

^1^ http://www.pasteur-guadeloupe.fr:8081/SITVITDemo/
