# Supplementary material for: Primary Bedaquiline Resistance Among Cases of Drug-Resistant Tuberculosis in Taiwan
Source: Front Microbiol. 2021 Oct 22;12:754249. doi: 10.3389/fmicb.2021.754249 (PMC8569445; doi:10.3389/fmicb.2021.754249)
Supplement: Supplementary file 1 [file Data_Sheet_1.pdf]

# *Supplementary Material*

Table S1. Phenotypic drug susceptibility patterns of 93 study isolates

| Drug resistance profiles | Total no. of isolates (%) | BDQ MIC | |
|---|---|---|---|
| | | ≥0.25 μg/mL (N=28) | ≤0.12 μg/mL (N=65) |
| **RR** | **12 (12.9)** | **5** | **7** |
| RIF only | 9 (9.7) | 5 | 4 |
| RIF+SM | 1 (1.1) | 0 | 1 |
| RIF+FQs | 1 (1.1) | 0 | 1 |
| RIF+PZA+FQs | 1 (1.1) | 0 | 1 |
| **MDR** | **45 (48.4)** | **18** | **27** |
| RIF+INH | 13 (14.0) | 4 | 9 |
| RIF+INH+EMB | 12 (12.9) | 6 | 6 |
| RIF+INH+PZA | 3 (3.2) | 2 | 1 |
| RIF+INH+SM | 4 (4.3) | 1 | 3 |
| RIF+INH+EMB+PZA | 4 (4.3) | 0 | 4 |
| RIF+INH+EMB+SM | 2 (2.2) | 1 | 1 |
| RIF+INH+EMB+PZA+SM | 7 (7.5) | 4 | 3 |
| **Pre-XDR** | **26 (28.0)** | **5** | **21** |
| RIF+INH+FQs | 2 (2.2) | 0 | 2 |
| RIF+INH+EMB+FQs | 4 (4.3) | 1 | 3 |
| RIF+INH+PZA+FQs | 3 (3.2) | 0 | 3 |
| RIF+INH+PZA+SLIDs | 1 (1.1) | 0 | 1 |
| RIF+INH+SM+FQs | 1 (1.1) | 0 | 1 |
| RIF+INH+SM+SLIDs | 1 (1.1) | 0 | 1 |
| RIF+INH+EMB+PZA+FQs | 3 (3.2) | 0 | 3 |
| RIF+INH+EMB+SM+FQs | 3 (3.2) | 0 | 3 |
| RIF+INH+EMB+PZA+SM+FQs | 8 (8.6) | 4 | 4 |
| **XDR** | **10 (10.8)** | **0** | **10** |
| RIF+INH+EMB+SM+FQs+SLIDs | 1 (1.1) | 0 | 1 |
| RIF+INH+PZA+SM+FQs+SLIDs | 2 (2.2) | 0 | 2 |
| RIF+INH+EMB+PZA+SM+FQs+SLIDs | 7 (7.5) | 0 | 7 |

RR, rifampicin-resistant; MDR, multidrug-resistant; pre-XDR, pre-extensively drug-resistant; XDR, extensively drug-resistant; RIF, rifampicin; INH, isoniazid; EMB, ethambutol; PZA, pyrazinamide; SM, streptomycin; SLIDs, second-line injectable drugs (including amikacin, capreomycin and kanamycin); FQs, fluoroquinolones (including moxifloxacin, levofloxacin, and ofloxacin).

Table S2. Characteristics and outcomes of 22 MGIT-bedaquiline-resistant TB cases.

| Case No. | DR type | Case category | Lineage | pDST (μg/mL) | | gDST^ | Outcome |
|---|---|---|---|---|---|---|---|
| | | | | MGIT (1.0) | MIC | *Rv0678* | |
| 1 | RR | New | H3 | R | 0.25 | Ins a 274–275 Fs (92stop) +WT | Cured |
| 2 | RR | New | T1 | R | 0.5 | Del 11–63 Fs (29stop) | Died |
| 3 | MDR | New | H3 | R | 0.12 | I80S | Cured |
| 4# | MDR | New | Beijing | R | 0.25 | Whole gene Del | Under treatment |
| 5 | MDR | New | Beijing | R* | 0.25 | WT | Cured |
| 6 | MDR | New | H3 | R | 0.25 | I80S | Cured |
| 7 | MDR | New | H3 | R* | 0.25 | WT | Cured |
| 8 | MDR | New | Beijing | R | 0.5 | L114P | Died |
| 9 | MDR | New | Beijing | R | 0.5 | Whole gene Del | Transferred out |
| 10 | MDR | New | EAI1-SOM | R | 0.5 | g-14a | Died |
| 11 | MDR | New | EAI2-Manila | R | 0.5 | S53P | Cured |
| 12 | MDR | New | T2 | R | 0.5 | A102V | Cured |
| 13# | MDR | New | Undefined | R | 0.5 | Ins g 181–182 Fs (80stop) | Cured |
| 14 | MDR | New | Undefined | R | 0.5 | F100Y | Cured |
| 15 | MDR | New | EAI2-Manila | R | 1 | S53P | Died |
| 16 | MDR | Previously treated | H3 | R | 0.25 | I80S | Treatment completed |
| 17 | MDR | Previously treated | Undefined | R | 0.5 | Ins g 181–182 Fs (80stop) | Cured |
| 18# | Pre-XDR | New | H | R* | 0.06 | WT | Cured |
| 19# | Pre-XDR | New | Beijing | R* | 0.12 | WT | Cured |
| 20# | Pre-XDR | New | T2 | R | 0.12 | C46Y | Cured |
| 21 | Pre-XDR | Previously treated | Beijing | R | 0.25 | L83P | Died |
| 22 | XDR | New | Beijing | R* | 0.06 | WT | Transferred out |

^All 22 cases had wild-type *atpE* and *pepQ* genes; #cases treated with bedaquiline; *borderline resistance.
WT, wild type; Del, deletion; Ins, insertion; Fs, frameshift mutation.

Table S3. Personalized regimens of 22 MGIT-bedaquiline-resistant TB cases.

| Case No. | Outcome | Drug resistance profile | Regimen | |
|---|---|---|---|---|
| | | | Active drugs^ | Inactive drugs# |
| 1 | Cured | RIF RFB BDQ | INH EMB PZA | RIF |
| 2 | Died | RIF RFB BDQ CFZ | INH EMB PZA MFX KM AMK CS PTO | RIF RFB |
| 3 | Cured | RIF INH EMB PZA RFB BDQ | MFX KM CS PTO | RIF INH EMB PZA |
| 4 | Under treatment | RIF INH EMB PZA RFB BDQ CFZ SM | MFX KM LZD CS PTO | RIF INH EMB PZA BDQ CFZ |
| 5 | Cured | RIF INH RFB BDQ SM | EMB PZA MFX LFX KM CFZ LZD PTO | RIF INH |
| 6 | Cured | RIF INH EMB BDQ | PZA MFX CM CFZ CS PTO SM TRD PAS | INH EMB RFB |
| 7 | Cured | RIF INH EMB RFB BDQ | PZA MFX KM CS PTO | RIF INH EMB |
| 8 | Died | RIF INH RFB BDQ | EMB PZA | RIF INH |
| 9 | Transferred out | RIF INH EMB PZA RFB MFX LFX BDQ CFZ SM | | |
| 10 | Died | RIF INH PZA BDQ | | |
| 11 | Cured | RIF INH EMB PZA BDQ CFZ SM | RFB MFX LFX KM LZD CS PTO DLM TRD | INH PZA CFZ |
| 12 | Cured | RIF INH BDQ CFZ | EMB PZA MFX KM PTO | RIF INH CFZ |
| 13 | Cured | RIF INH EMB RFB BDQ CFZ | PZA MFX KM LZD CS PTO | RIF INH EMB BDQ CFZ |
| 14 | Cured | RIF INH RFB BDQ CFZ | PZA MFX LFX KM CS PTO TRD | RIF INH EMB |
| 15 | Died | RIF INH EMB BDQ SM | PZA | RIF INH EMB |
| 16 | Treatment completed | RIF INH EMB BDQ | PZA MFX KM CS SM ETO | RIF INH EMB |
| 17 | Cured | RIF INH PZA RFB BDQ | EMB MFX CM CS SM ETO PAS | RIF INH PZA |
| 18 | Cured | RIF INH RFB KM AMK CM BDQ SM | EMB PZA MFX LFX CFZ LZD TRD | RIF INH BDQ SM |
| 19 | Cured | RIF INH EMB RFB MFX LFX BDQ | PZA KM CFZ CS PAS | EMB BDQ |
| 20 | Cured | RIF INH RFB MFX LFX BDQ CFZ SM | EMB PZA KM CM LZD CS PTO PAS | RIF INH MFX BDQ |
| 21 | Died | RIF INH EMB PZA RFB MFX BDQ SM | KM | RIF INH EMB PZA MFX |
| 22 | Transferred out | RIF INH EMB PZA RFB MFX LFX KM AMK CM BDQ SM | | RIF INH EMB PZA |

RIF, rifampicin; INH, isoniazid; EMB, ethambutol; PZA, pyrazinamide; RFB, Rifabutin; MFX, moxifloxacin; LFX, levofloxacin; KM, kanamycin; AMK, amikacin; CM, capreomycin; BDQ, bedaquiline; LZD, linezolid; CFZ, clofazimine; CS, cycloserine; PTO, prothionamide; DLM, delamanid; SM, streptomycin; TRD, terizidone; ETO, ethionamide; PAS, para-aminosalicylic acid.

^Active drugs are defined as cases susceptible to the drugs used for treatment and #inactive drugs as cases resistant to the drugs.
